# LC-ESI-QTOF-MS/MS Analysis, Cytotoxic, Antiviral, Antioxidant, and Enzyme Inhibitory Properties of Four Extracts of *Geranium pyrenaicum* Burm. f.: A Good Gift from the Natural Treasure

**DOI:** 10.3390/ijms22147621

**Published:** 2021-07-16

**Authors:** Łukasz Świątek, Elwira Sieniawska, Kouadio Ibrahime Sinan, Magdalena Maciejewska-Turska, Anastazja Boguszewska, Małgorzata Polz-Dacewicz, Ismail Senkardes, Gokalp Ozmen Guler, Nabeelah Bibi Sadeer, Mohamad Fawzi Mahomoodally, Gokhan Zengin

**Affiliations:** 1 Department of Virology with SARS Laboratory, Medical University of Lublin, Chodzki 1, 20-093 Lublin, Poland; anastazjaboguszewska@umlub.pl (A.B.); malgorzata.polz-dacewicz@umlub.pl (M.P.-D.); 2 Department of Pharmacognosy, Medical University of Lublin, Chodzki 1, 20-093 Lublin, Poland; esieniawska@pharmacognosy.org (E.S.); magdalena.maciejewska@umlub.pl (M.M.-T.); 3 Physiology and Biochemistry Research Laboratory, Department of Biology, Science Faculty, Selcuk University, Konya 42130, Turkey; sinankouadio@gmail.com; 4 Department of Pharmaceutical Botany, Faculty of Pharmacy, Marmara University, Istanbul 34854, Turkey; isenkardes@marmara.edu.tr; 5 Department of Biological Education, Ahmet Kelesoglu Education Faculty, Necmettin Erbakan University, Konya 42090, Turkey; gguler@erbakan.edu.tr; 6 Department of Health Sciences, Faculty of Medicine and Health Sciences, University of Mauritius, Réduit 80837, Mauritius; nabeelah.sadeer1@umail.uom.ac.mu (N.B.S.); f.mahomoodally@uom.ac.mu (M.F.M.)

**Keywords:** *Geranium*, antioxidants, polyphenols, enzymes, HSV-1, cytotoxicity

## Abstract

This study focused on the biological evaluation and chemical characterization of *Geranium pyrenaicum* Burm. f. Different solvent extracts (hexane, ethyl acetate, methanol, and water extracts) were prepared. The phytochemical profile, antioxidant, and enzyme inhibitory activity were investigated. Cytotoxicity was assessed using VERO, FaDu, HeLa and RKO cells. The antiviral activity was carried out against HSV-1 (Herpes simplex virus 1) propagated in VERO cell line. The aqueous extract, possessing high phenolic content (170.50 mg gallic acid equivalent/g extract), showed the highest reducing capacity (613.27 and 364.10 mg Trolox equivalent/g extract, for cupric reducing antioxidant capacity and ferric reducing antioxidant power, respectively), radical scavenging potential (469.82 mg Trolox equivalent/g extract, against 2,2′-azino-bis(3-ethylbenzothiazoline-6-sulfonic acid)), metal chelating ability (52.39 mg ethylenediaminetetraacetic acid equivalent/g extract) and total antioxidant capacity (3.15 mmol Trolox equivalent/g extract). Liquid chromatography-electrospray ionization-quadrupole time-of-flight-mass spectrometry (LC-ESI-QTOF-MS/MS) alloved to tentatively identify a total of 56 compounds in the extracts, including ellagitannins, gallic acid and galloyl derivatives amongst others. The ethyl acetate extracts substantially depressed cholinesterase enzymes (4.49 and 12.26 mg galantamine equivalent/g extract against AChE and BChE, respectively) and α-amylase enzyme (1.04 mmol acarbose equivalent/g extract). On the other hand, the methanolic extract inhibited tyrosinase (121.42 mg kojic acid equivalent/g extract) and α-glucosidase (2.39 mmol acarbose equivalent/g extract) activities. The highest selectivity towards all cancer cell lines (SI 4.5–10.8) was observed with aqueous extract with the FaDu cells being the most sensitive (CC_50_ 40.22 µg/mL). It can be concluded that the presence of certain bioactive antiviral molecules may be related to the high anti HSV-1 activity of the methanolic extract. This work has generated vital scientific data on this medicinal plant, which is a prospective candidate for the creation of innovative phyto-pharmaceuticals.

## 1. Introduction

The investigation of phytochemical profiles and biological properties of medicinal plants is an important step in determining whether the plant in question can reach production systems and use its bioactive compounds in the pharmaceutical industry. In this line of thought, our aim with this research paper is thus to screen a medicinally important plant *Geranium pyrenaicum* Burm. f. (*G. pyrenaicum*) in terms of their biological properties (antioxidant and anti-enzymatic properties) and phytochemicals. Plants from the *Geranium* genus, which comprises about 250 species, have been used since ancient times in the practice of traditional medicines throughout the world [1]. Several studies revealed antimicrobial activity of essential oils isolated from different parts of *Geranium* species against several microorganisms, which could prospect their potential use as natural antimicrobial agents. Also, antimicrobial properties along with observed antioxidant effects, indicate their potential application as natural food preservatives [1]. From *G. pyrenaicum*, which exhibits antileishmanial activity, a new glycosylate flavonoid, 3-*O*-(2″,3″-di-*O*-galloyl)-*O*-d-glucopyranoside of kaempferol was identified [2].

Plant species belonging to the Geraniaceae family were shown to possess variable cytotoxicity towards cancer and normal cell lines. Nunes et al. studied the anti-proliferative properties of *Geranium purpureum* Vil. and didn’t find any significant activity towards BJ (normal adherent human skin fibroblasts) and Hep G2 (hepatocellular carcinoma) cells [3]. The *Geranium macrorrhizum* leaves and roots methanolic extracts showed moderate cytotoxicity towards human leukemia cell lines CCRF-CEM and CEM/ADR 5000, with CC_50_ values of 22.4 and 98.3 µg/mL for CCRF-CEM and 112.3 and 154.2 µg/mL for CEM/ADR 5000 cell, respectively. Lower cytotoxicity towards CEM/ADR 5000 cells can be due to the overexpression of ABC (ATP Binding Cassette) transporters, such as P-glycoprotein (Pgp), which are responsible for the extrusion process of xenobiotics, and thus are often responsible for multidrug resistance (MDR) [4]. The anticancer potential of *Geranium dielsianum* ethanolic extract was tested towards a panel of cancer cell lines (M-14, DU-145, H-460, HT-29, MCF-7, and K562) and normal mouse embryo cells (3T3). The values of IC_50_ (50% inhibitory concentration) obtained for cancer cells were in the range of 75.13–196.54 µg/mL, whereas, in the case of the mouse embryo normal cell the reported IC_50_ value was 58.12 µg/mL, indicating no cancer selectivity [5]. No significant cytotoxicity towards cell lines was found for *Geranium krameri* towards B16-F10 (*Mus musculus* skin melanoma) [6], *Geranium koreanum* Kom. tested on Raw 264.7 (*Mus musculus* macrophage) [7], *Geranium stepporum* and *Geranium psilostemon* towards KB (subline of the KERATIN-forming HeLa cell line) [8], whereas, *Geranium sibiricum* L. methanolic extract was reported to increase the proliferation rate of human dermal papilla cells (hDPCs) by 132.7% [9]. 

Herpes simplex virus 1 (HSV-1, HHV-1, aka Human herpesvirus type 1) is an enveloped double-stranded DNA virus belonging to the Herpesviridae family. Currently, eight human herpesviruses have been identified and divided into three subfamilies: Alphaherpesvirinae (HSV-1, HSV-2, and HHV-3 (VZV, varicella zoster virus); Betaherpesvirinae (HHV-4 (CMV, human cytomegalovirus), HHV-6, and HHV-7); and Gammaherpesvirinae (HHV-5 (EBV, Epstein–Barr virus) and HHV-8 (KSHV, Kaposi’s sarcoma-associated herpesvirus)). Primary infection with HSV-1 usually occurs during the first two decades of life, and subsequently, the virus establishes lifelong latency in dorsal root ganglia [10,11]. Reactivation of latent HSV-1 is often asymptomatic, but may also lead to severe, even life-threatening diseases (ex. encephalitis), especially among immune-suppressed patients [11]. The *Geranium sanguineum* L. was shown to possess antiviral activity towards the Influenza virus [12,13], HSV-1 and HSV-2 [14], whereas, *Geranium carolinianum* L. was found to inhibit the replication of hepatitis B virus (HBV) [15].

Despite various bioactivities of *Geranium* species are known, there are no data regarding enzymatic inhibitory effects of *G. pyrenaicum*. We, therefore, designed this study to validate the antioxidant and enzymatic inhibitory activities of *G. pyrenaicum*, which is an underexplored plant. To the best of our knowledge, there is currently a paucity of information in the scientific literature regarding the inhibitory effects of *G. pyrenaicum* extracts against the key clinical enzymes namely cholinesterase, tyrosinase, α-glucosidase and α-amylase. We also sought to screen the plant for its phytochemical composition. Furthermore, there are no reports on the cytotoxicity and antiviral activity of *G. pyrenaicum* that is why we have decided to evaluate these biological activities of this plant species. The evaluation of *G. pyrenaicum* cytotoxicity was carried out in vitro on normal VERO (ATCC, No. CCL-81) cells and cancer cell lines—FaDu (ATCC, HTB-43, hypopharyngeal squamous cell carcinoma), HeLa (ECACC No. 93021013, cervical adenocarcinoma) and RKO (ATCC, CRL-2577, colon carcinoma), using a protocol based on 3-(4,5-dimethylthiazol-2-yl)-2,5-diphenyltetrazolium bromide (MTT). The evaluation of the antiviral activity of *G. pyrenaicum* extracts was carried out against HSV-1 (ATCC, No. VR-260) propagated in VERO cell line. The antiviral assays included the influence of tested extracts on the formation of HSV-1 induced cytopathic effect, end-point virus titration for infectious titer, and the Real-Time PCR analysis for viral load. It is anticipated that the presented results could fill in the research gap and subsequently could open new research avenues, particularly with respect to therapeutic bioproduct development. 

## 2. Results and Discussion

### 2.1. Bioactive Compounds

In the present study, standard in vitro spectrophotometric analyses was conducted to determine total phenolic (TPC) and flavonoid contents (TFC) of the hexane, ethyl acetate, methanolic and aqueous extracts of *G. pyrenaicum*. The results are given in Table 1. As it can be observed from Table 1, the highest TPC and TFC were obtained from aqueous (170.50 mg GAE/g) and ethyl acetate (43.95 mg RE/g) extracts, respectively while the lowest TPC and TFC were from hexane (25.03 mg GAE/g) and aqueous (25.11 mg RE/g) extracts, respectively. 

However, spectrophotometric studies can only offer information about the content of phenolic compounds in herbal extracts without having precise phytochemical composition. Therefore, the extracts were subjected to liquid chromatography coupled to mass spectrometry (LC-ESI-QTOF-MS/MS) to assess the detailed profile of *G. pyrenaicum*. The tentatively identified compounds from the extracts were summarized in Table 2.

In the present work, the analysis of the chemical composition of four selected *Geranium* extracts was carried out using LC-ESI-QTOF-MS/MS method in negative ionization mode. A total of 56 molecules presented in Table 2 and in Figure S1 were tentatively identified. The chemical profile of the studied samples was strongly dependent on the extraction solvent. The detected polyphenols, mainly ellagitannins, were the most represented in samples extracted with polar solvents, primarily methanol, indicating this alcohol as the most efficient extraction solvent. 

#### 2.1.1. Ellagitannins

The prevailing class of polyphenols found in *Geranium* samples were hydrolysable tannins esterified with hexahydroxydiphenoyl group (HHDP) and glucose. Most of these constituents comprised of galloyl and HHDP moiety, thus in their MS/MS spectra, the typical neutral elimination of galloyl (−152 Da), gallic acid (−170 Da), galloylglucose (−332 Da), HHDP (−302 Da) or HHDP-glucose residue (−482 Da) was observed [42]. Additionally, formation of a key fragment ion at *m*/*z* 301, resulted from lactonization of the HHDP ester group into more stable ellagic acid structure was predominant in these constituents [42]. The classification of compounds into ellagic acid or quercetin-based conjugates was made on the basis of typical for these structures fragments ions appearing in MS/MS spectra. Thus, fragment ions at *m*/*z* 283 [M-H-H_2_O]^−^, 229 [M-H-CO_2_-CO]^−^, 201 [M-H-CO_2_-CO-CO]^−^, 185 [M-H-2CO_2_-CO]^−^, formed from precursor ion at *m*/*z* 301, evidenced the presence of ellagic acid-based conjugates and were distinguished from quercetin-based structures for which apart from fragment ions due to the loss of small molecules, the typical ions at *m*/*z* 179 and especially at *m*/*z* 151 were identified as a result of rDA cleavage of C-ring [27,38,43].

Free ellagic acid (39) was tentatively identified on the basis of aforementioned fragments ions at 24.240 min in both aqueous and methanolic extracts. Compounds 24 (tR = 14.299 min) and 29 (tR = 19.130 min) with an [M-H]^−^, ion at *m*/*z* 633 and identical fragmentation pattern were supposed to be isomers. The major fragment ions observed at *m*/*z* 463 [M-H-170]^−^, and 301 [M-H-170-162]^−^, were consistent with the loss of gallic acid and one glucose group, bonded to HHDP unit. Additionally, fragments ions at *m*/*z* 301, 275 and at *m*/*z* 169, 125 were consistent with previously reported in *Phyllanthus* sp. HDDP-galloyl-glucose isomers, tentatively identified as corilagin (24) and isocorilagin (29), according to their elution behaviour mentioned by Yang, et al. [44]. Similar fragmentation behaviour was observed for compound 31, tentatively identified as geraniin, on the basis of the precursor peak at *m*/*z* 951 and prominent fragmentation ions at *m*/*z* 463, 301 and 169 [45]. While the successive elimination of H_2_O from [M-H]^−^, ion matched with fragment ions at *m*/*z* 933 and 915 found in MS/MS spectrum [36]. This compound was detected only in methanolic extract in 20.417 min in low intensity. 

Two isomers 16 and 21 yielding an identical precursor ion [M-H]^−^, at *m*/*z* 783 were observed in MS spectra of methanolic sample alone. The MS/MS analysis of their fragmentation pattern revealed two fragment ions at *m*/*z* 481 [M-H-302]^−^, and 301 [M-H-482]^−^, corresponding to the deprotonated HHDP-glucose and ellagic acid, respectively. Ions at *m*/*z* 275 and 249 resulted from decarboxylation of HHDP unit [27,46]. The separate elimination of HHDP and HHDP-glucose moiety from precursor ion, indicate on two isomers of di-HHDP-glucose, presumably with a pedunculagin-like structure [27,28,29].

A slightly different fragmentation was observed for compound 30 tentatively identified in both aqueous and methanolic samples as di-galloyl-HHDP-glucoside. The fragment ion at *m*/*z* 633 was detected as a result of the elimination of galloyl moiety from precursor ion (*m*/*z* 785). The formation of ion at *m*/*z* 483 derived from di-galloyl-glucoside [46], while ions at 301 and 169 corresponded to ellagic and gallic acids, respectively. In the view of the above, compound 30 comprised two galloyl units linked to HHPD group via one glucose residue corresponding to Tellimagrandin I structure [30,35].

Compounds 27 and 28 eluted in 17.761 and 18.026 min in aqueous and methanolic extracts, showed identical fragmentation pattern. Compound 27 with molecular formula of C_12_H_8_O_6_ and an abundant product ion [M-H]^−^, at *m*/*z* 247.0244 generated fragment ions at *m*/*z* 219 and 191, resulted from successive loss of CO unit. Similarly, in case of compound 28 the major fragment ion at *m*/*z* 247 was formed via decarboxylation of its product ion at *m*/*z* 291. The further fragmentation was consistent with those of the aforementioned for compound 27. Comparing with literature data compounds 27 and 28 were tentatively identified as brevifolin and brevifolincarboxylic acid, respectively, while compounds 26 (methyl brevifolincarboxylate derivative), 32 (methyl brevifolincarboxylate isomer 1) and 35 (methyl brevifolincarboxylate isomer 2) as methylated derivatives of brevifolincarboxylic acid [33,35]

The analysis of the fragmentation pattern of compounds 46 and 47, allowed only their preliminary identification. Due to the fragment ions generated in their MS/MS spectra at *m*/*z* 827 [M-H-170]^−^, 301 [M-H-170]^−^, 525 [M-H-170-302]^−^, 363 [M-H-170-302-162]^−^, for compound 46 and ions at *m*/*z* 599 [M-H-152]^−^, and 313 [M-H-152-134-152]^−^, for compound 47, they were tentatively assigned as derivatives of HHDP-galloyl-glucoside and HHDP-galloyl, respectively.

The compound 33, which generated precursor ion [M-H]^−^, at *m*/*z* 395 with molecular formula of C_17_H_16_O_11_, was detected only in methanolic extract (22.134 min). The fragment ions at *m*/*z* 363 and 319 were identified as a result of the elimination of methanol and carbon dioxide from molecular ion, respectively. Due to the lack of sufficient data compound 33 was assigned as dehydrochebulic acid trimethyl ester, previously reported by Geethangili and Ding [37] in *Phyllantus urinaria* and in the twigs and leaves extracts of African medicinal plant, *Flueggea virosa* by Chao, et al. [47].

#### 2.1.2. Gallic Acid and Galloyl Derivatives

The gallic acid (8) was identified in this study in 4.049 min with [M-H]^−^, at *m*/*z* 169 and fragment ion at *m*/*z* 125, corresponding to the characteristic loss of CO_2_ unit. Aforementioned fragment ions found in MS/MS spectra of compounds 6, 7, 9, 13, 15, 25 confirmed the existence of gallic acid in their structure. The simplest gallotaninnin, glucogallic acid (7), with molecular formula of C_13_H_16_O_10_ and precursor [M-H]^−^, ion at *m*/*z* 331 was found in almost all, besides water extracts. The prominent fragment ions at *m*/*z* 169, 125 corresponded to the gallic acid fragmentation, while fragments at *m*/*z* 313, 271, 241 and 211 originated from cross-ring fragmentation of a glucose molecule and were consistent with literature data [46,48]. Similar product ions were observed in the MS/MS spectra of compound 13 (*m*/*z* 483) identified in two polar (methanolic and aqueous) extracts. The additional 152 unit in its structure suggested the gallic acid O-(6-galloylglucoside) or di-galloyl-hexoside, previously reported in different *Geranium* sp. [23,24,46]. Tentative identification of other galloyl esters was based on their fragmentation pattern compared to that available in literature findings and production of dominant product ions at *m*/*z* 191 and 173, corresponding to deprotonated quinic acid (6, 9, 25) or shikimic acid (15), respectively [27]. The glicerol gallate (11) was tentatively identified only in methanolic extract on the basis of HMDB database. The MS/MS spectra of compound 20 with [M-H]^−^, product ion at *m*/*z* 183 generated dominant radical ions at *m*/*z* 168 and 124. The neutral loss of 15 Da suggested the methoxyled form, while abundant fragments ions on gallic acid structure, thus compound 20, determined in all studied extracts, was assigned as methyl gallate [25]. 

#### 2.1.3. Other Polyphenols

Compounds eluted between 23.773 and 25.640 min were classified to the flavonols, in particular to quercetin (37, 38, 41, 42, 45) or kaempferol based (43, 44, 49) derivatives, identified on the basis of their abundant fragment ions appearing at *m*/*z* 301 for quercetin and at *m*/*z* 285 for kaempferol.

Quercetin (48) was identified in all studies extracts in 30.914 min, on the basis of a typical for this structure fragment ions mentioned above. For compounds 37, 38 and 41 the typical for hexosides (both for glucosides and galactosides) and glucoronides neutral loss of 162 and 176 Da from precursor ion was observed in MS/MS spectra, respectively. Additionally, the detection of quercetin-like ions at *m*/*z* 179 and 151 enabled their tentative identification as quercetin-*O*-hexoside derivative (37), quercetin-*O*-hexoside (38), quercetin-*O*-glucuronide (41). In MS/MS spectra of compounds 42 and 45 additional fragments ions at *m*/*z* 169 and *m*/*z* 125 corresponding to a galloyl residue indicated the presence of guercetin-*O*-(galloyl)-hexoside (42) and quercetin-di-galloyl-hexoside (45) previously reported by Li and Seeram [40] and Gu, Yang, Bakri, Chen, Xin and Aisa [46]. Similarily, compounds 43, 44, 49 primarily found in methanolic extracts were associated with kaempferol aglycone. On the basis of literature findings and available databases they were tentatively identified as kaempferol-*O*-galloyl- hexoside (43), kaempferol-*O*-hexoside (44) and kaempferol-3-*O*-rutinoside (49). In case of compound 50, detected in all tested samples, the intense fragment ion in its MS/MS spectra at *m*/*z* 133 formed though rDA cleavage of precursor ion [M-H]^−^, at *m*/*z* 285 indicated luteolin rather than kaempferol structure.

The hydroxycinnamic acids were represented mainly by caffeic acid derivatives. By comparison of their fragmentation pattern with those reported previously we were able to assign them as: caffeic acid glucoside (1), dihydrocaffeic acid (17) and caffeoylmalic acid (22). Compounds eluted after 31 min were assigned to fatty acid group (51, 52, 53, 55, 56), for which chemical structure elucidation was impossible solely with the ESI-MS method.

### 2.2. Antioxidant Effects

The role of oxidative stress in the initiation and/or progression of human ailments lends support to the systemic antioxidant assessment of plant extracts under investigation. Antioxidants can work through a variety of mechanisms, including hydrogen atom transfer, single electron transfer, and transition metal chelation [49]. In this study, a battery antioxidant assays were used to obtain a comprehensive understanding of the antioxidant activities of the prepared *G. pyrenaicum* extracts namely 2,2-diphenyl-1-picrylhydrazyl (DPPH), 2,2′-azino-bis (3-ethylbenzothiazoline-6-sulfonic acid) (ABTS), ferric ion reducing antioxidant power (FRAP), cupric reducing antioxidant capacity (CUPRAC), metal chelating and total antioxidant capacity (phosphomolybdenum). Each assay has their own strengths and limitations as detailed elsewhere. Results are given in Table 1 and Table 3. 

As presented in Table 3, the highest and lowest ABTS activity was recorded in the aqueous extract (469.82 mg TE/g, for ABTS) and hexane extract (6.02 mg TE/g) of *G. pyrenaicum*, respectively. The antioxidant activity of the aqueous and hexane extracts correlated with the content of phenolic compounds which showed that the aqueous extract contained the highest TPC while the hexane extract yielded the lowest phenolic content (Table 1). Thus, it is suggested that the ABTS activity was directly linked with phenolic compounds present. 

Furthermore, the radical scavenging abilities of the tested extracts were evaluated by DPPH assay and the best DPPH radical scavenging ability was provided by the methanolic extract with the value of 199.26 mg TE/g. Hexane extract was also not active on DPPH radical. As above-mentioned, the aqueous extract was noted as the most active extract in ABTS assay. The difference could be explained with the nature of these radicals. For example, ABTS radical could be performed in hydrophilic and lipophilic media, while DPPH could be just utilized in lipophilic one [49]. In this sense, different molecules may be acted in the assays.

The antioxidant potential of the extracts was further assessed in terms of reducing power using the CUPRAC and FRAP methods. With CUPRAC assay, 1 g of dried aqueous extract exhibited the most potent reducing activity (613.27 mg TE/g) followed by methanolic (514.79 mg TE/g), ethyl acetate (89.59 mg TE/g) and hexane (64.18 mg TE/g) extracts. In addition, a similar trend was observed with FRAP assay, revealing the most active extract to be the aqueous extract (364.10 mg TE/g) followed by methanolic (294.54 mg TE/g), ethyl acetate (40.43 mg TE/g) and hexane (30.68 mg TE/g) extracts. A good correlation between the results of CUPRAC and FRAP assays was also noted by previous studies [49,50,51].

Secondary metabolites are recognized to have considerable antioxidant characteristics, not only because of their potential to give electrons, but also because they chelate transition metals [52]. Similar to DPPH, ABTS, CUPRAC and FRAP assays, among the tested extracts, the aqueous extract (52.39 mg EDTAE/g) showed the most effective chelating ability. The total antioxidant capacity of the tested samples was also determined. The latter test is based on antioxidants reducing Mo (VI) to Mo (V), resulting in the formation of a green complex in acidic conditions [49]. The aqueous extract showed the highest total antioxidant capacity (3.15 mmol TE/g) as shown in Table 1.

Several scientists have demonstrated that ellagitannins compounds, abounded in the extracts, are frequently involved in antioxidant ability of plant extracts [53,54]. The in vitro surveys on human cells culture have highlighted the protective role of ellagic acid in suppressing oxidative stress through the activation of some specific genes of the antioxidant defence system [55]. The authors suggest that the presence of two pairs of hydroxyl groups as well as two aromatic rings contribute greatly to the antioxidant potential of ellagic acid. Nandini and Naik [53] showed that coraligin significantly ameliorate oxidative stress in the streptozotocin-induced rats by increasing catalase, superoxide dismutase activity and reduced glutathione level. Study focused on pedunculagin and tellimagrandin I isolated from *Eucalyptus* leaves revealed that both molecules have highest antioxidant activity than trolox and BHA in both DPPH and ABTS assays [56]. Well over, the authors reported that both compounds exhibited excellent cellular antioxidant activity in cell-based assays. Furthermore, phytochemicals identified in the tested extracts may have acted in synergy resulting in such outcomes. On the other hand, hexane extracts displayed the lowest antioxidant properties with all assays corroborating with its low TPC.

### 2.3. Enzyme Inhibitory Activities

Enzymes are very effective natural catalysts for all biochemical processes found in nature. A healthy bodily system is maintained when enzymes are present at a normal level in the body. Overexpression of enzymes, on the other hand, might result in aberrant biological processes, which can lead to clinical consequences. Due to all the different negative effects of synthetic medications, scientists are looking into the enzyme inhibitory potential of plants. In the present study, the ability of *G. pyrenaicum* extracts to modulate the activity of enzymes related to Alzheimer’s disease [acetylcholinesterase (AChE) and butyrylcholinesterase (BChE)], diabetes type 2 (α-amylase and α-glucosidase), and skin hyperpigmentation (tyrosinase) was investigated and the results are presented in Table 4.

Due to the existence of enzymes in the human body causes illness aetiology, blocking these enzymes can be a helpful technique in illness treatment. Cholinesterase inhibitors, for example, are medications that block the breakdown of acetylcholine, a major neurotransmitter of the central nervous system that, if present in high levels, can induce neurodegenerative illnesses such as Alzheimer’s and Parkinson’s diseases. In our study, we screened the different extracts for possible anti-cholinesterase activity. High anti-AChE (4.49 mg GALAE/g) is recorded with the ethyl acetate and methanol extracts while strong anti-BChE (12.26 mg GALAE/g) activities is provided by the ethyl acetate extract. In a previous study, it was reported that quercetin, a compound also reported present in the ethyl acetate extract herein, exhibited potential anti-AChE activity (76.2%) [57]. Similarly, [58] indicated that luteolin shows highest anti-AChE and anti-BChE activity at 1.0 mmol/mL and 0.2mmol/mL respectively. While, gallic acid was shown to improve memory by improving synaptic strength, decreasing the size of plaque Aβ in the brain and increasing the acetylcholine level via the reduction of cholinesterase enzymes activity [59]. Commercial standards of corilagin, a compounds abounded in methanol extract, were reported to have AChE inhibitory effects with IC_50_ values of 0.72 ± 0.03 mM [60]. In addition, Abd El-Aziz, et al. [61] showed that ellagic acid exert competitive inhibition of AChE with IC_50_ of 1.927 mg/mL. However, the phytochemical profiles presented here are qualitative only. Therefore, without quantitative information on the identified compounds, it is difficult to determine which compounds are bringing the largest share in the anticholinesterase activity.

α-Amylase and α-glucosidase inhibitors delay the breakdown of carbohydrates in the small intestine and as a consequent decrease the post-prandial blood glucose level which is considered as an important treatment strategy to manage blood glucose level in type 2 diabetic patients [62]. The ethyl acetate extract was also observed to substantially depress α-amylase activity (1.04 mmol ACAE/g). The extract was shown to yield the highest TFC (Table 1). However, it was the methanolic extract that demonstrated the highest anti-glucosidase (2.39 mmol ACAE/g). From Table 4, the aqueous extract of *G. pyrenaicum* was found to be the least potent α-amylase (0.21 mmol ACAE/g) and α-glucosidase (2.04 mmol ACAE/g) inhibitors, despite this extract yielded the highest TPC. Furthermore, by referring to many reports, we presume that certain molecules i.e., corilagin, tellimagrandin I, ellagic acid, quercetin, gallic acid might be active principles responsible for the anti-amylase and/or anti-glucosidase actions [53,63]

Tyrosinase inhibitors help to protect the skin and prevent skin hyperpigmentation. They are greatly warranted by the pharmaceutical and cosmeceutical industries [64]. The methanolic extract displayed the strongest anti-tyrosinase (121.42 mg KAE/g) activities. It is worthy to note that this extract did not possess high TPC and TFC and thus such outcomes can be explained by the synergistic effect which is a well-known phenomenon, especially in pharmaceutical industries whereby a combination of compounds is preferred to produce drugs with the desired effect [65]. Nevertheless, these data need to be investigated further to determine whether the hypothesis of synergy holds true. Besides some molecules present in the methanol extract may be responsible for the observed activity. In an earlier study, it was indicated that pedunculagin shows 27% mild inhibitory effect toward tyrosinase activity [66] In silico study focused on the major compounds of *Geranium species* showed that ellagic acid exhibited good molecular linked with the active site of tyroninase through a connection with two Cu atoms being at the center of the enzyme [67]. In addition, the authors showed that corilagin and geraniin are most active inhibitors of tyrosinase enzyme. 

### 2.4. Cytotoxic Evaluation

**Figure 1:**
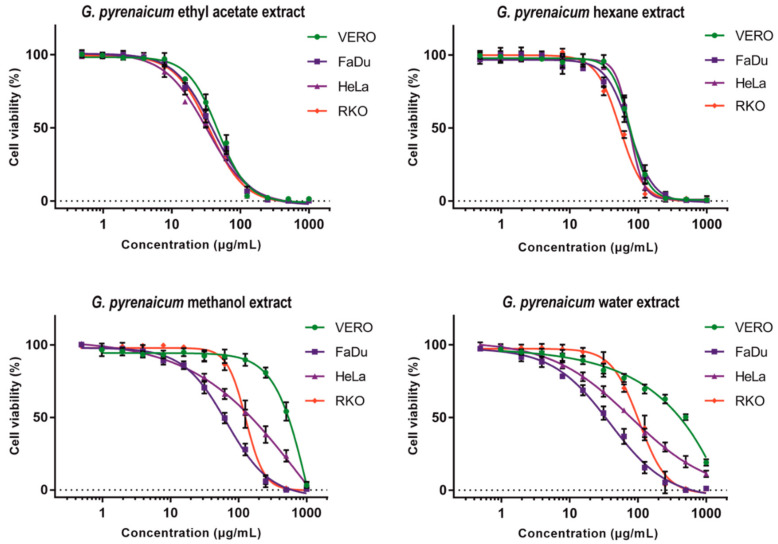
Dose–response influence of *Geranium pyrenaicum* extracts on cell lines.

Results of cytotoxicity evaluation are presented in Table 5. The *Geranium pyrenaicum* hexane extract (GP-H) showed comparable CC_50_ values on all tested cell lines. The dose response curves of GP-H presented on Figure 1 are similar for VERO, FaDu and HeLa. In case of RKO, GP-H showed higher toxicity with CC_50_ of 53.72 µg/mL and SI of 1.4 but this difference was not statistically significant (*p* > 0.05). Interestingly, the *Geranium pyrenaicum* ethyl acetate extract (GP-EA) showed lower CC_50_ values on all tested cancer cell lines in comparison to VERO cells with selectivity index (SI) 1.2–1.4, however, those differences were also not significant. With the increasing polarity of solvent used for extraction an increase of SI could be observed. In case of *Geranium pyrenaicum* methanolic extract (GP-ME) a significant anticancer activity on all cell lines could be observed (SI 3.6–7.2) and the highest activity (CC_50_ 66.92 µg/mL) and selectivity found on FaDu cells. The highest selectivity towards all cancer cell lines (SI 4.5–10.8) was observed for *Geranium pyrenaicum* aqueous extract with the FaDu cells being the most sensitive (CC_50_ 40.22 µg/mL).

The *Geranium maculatum* ethanolic extract was reported to possess moderate (IG_50_ (50% inhibitory growth) 60.2 µg/mL) on MDA-MB-231 (human breast cancer) cells [68], which is similar to the activity we have observed for *Geranium pyrenaicum* methanol extract on FaDu cells (CC_50_ 66.92 µg/mL). Graça, Barros, Calhelha, Dias, Carvalho, Santos-Buelga, Santos and Ferreira [24] reported the cytotoxicity of organic (n-hexane, dichloromethane, ethyl acetate, acetone and methanolic) and aqueous (infusion and decoction) extracts from *Geranium robertianum* L. towards MCF-7 (breast adenocarcinoma), NCI-H460 (non-small cell lung cancer), HeLa and HepG2 (hepatocellular carcinoma) cancer cell lines, as well as hepatotoxicity against non-tumor porcine liver primary cells (PLP2). The acetone extract exerted highest cytotoxicity against all tumor (GI_50_—50% of growth inhibition, 57–71 µg/mL) and non-tumor (GI_50_ 176 µg/mL) cell lines [24]. Interestingly, cytotoxicity of *Geranium robertianum* n-hexane extract was noticeably lower towards cancer cells (GI_50_ 151–179 µg/mL) [24] than we have observed (Table 5) for *Geranium pyrenaicum* n-hexane extract (CC_50_ 53.72–75.46 µg/mL), in case of HeLa cells, this difference was 162 vs. 73.28 µg/mL, respectively. Moreover, *Geranium pyrenaicum* ethyl acetate extract was more toxic to cancer cells than *Geranium robertianum* ethyl acetate extract, and the difference was especially marked in case of HeLa cells, 32.34 vs. 217 µg/mL, respectively. *Geranium robertianum* aqueous extracts (infusion, decoction) showed variable cytotoxicity against cancer cells (GI_50_ 45.68–380 µg/mL) [24], whereas, we have observed consistent anticancer activity of *Geranium pyrenaicum* aqueous extract towards all tested cell lines with high selectivity (Table 5). In another paper based on similar study design, Graça, Dias, Barros, Calhelha, Santos and Ferreira [23] reported the cytotoxicity of *Geranium molle* L. extracts towards the same cancer panel and PLP2 cells. The *Geranium molle* L. acetone extract proved to possess the highest toxicity towards cancer (GI_50_ 50–85 µg/mL) and PLP2 (GI_50_ 191 µg/mL) cells, and the results were in accordance with those observed for *Geranium robertianum*. Conversely, *Geranium molle* L. ethyl acetate and methanol extracts exerted significantly higher cytotoxicity towards most of the cancer cell lines than the extract obtained using the same solvents from *Geranium robertianum* [24]. The influence of *Geranium robertianum* and *Geranium molle* on PLP2 proved low hepatoxicity (GI_50_ > 400 µg/mL) for infusion, decoction, n-hexane and dichloromethane extracts, whereas, for acetone, ethyl acetate and methanolic it was markedly higher with GI_50_ of 176, 282 and 290 µg/mL, respectively [23,24]. Interestingly, in case of *Geranium pyrenaicum*, the methanolic and aqueous extracts showed lower toxicity towards non-cancerous VERO cells than hexane and ethyl acetate extracts (Table 5).

### 2.5. Antiviral Activity

**Figure 2:**
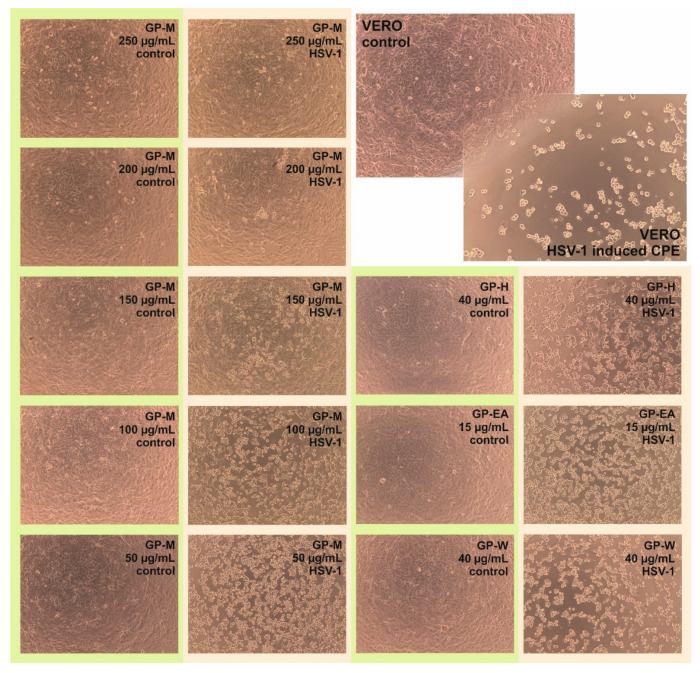
Influence of *Geranium pyrenaicum* extracts on HSV-1 induced CPE formation in VERO cells.

**Figure 3:**
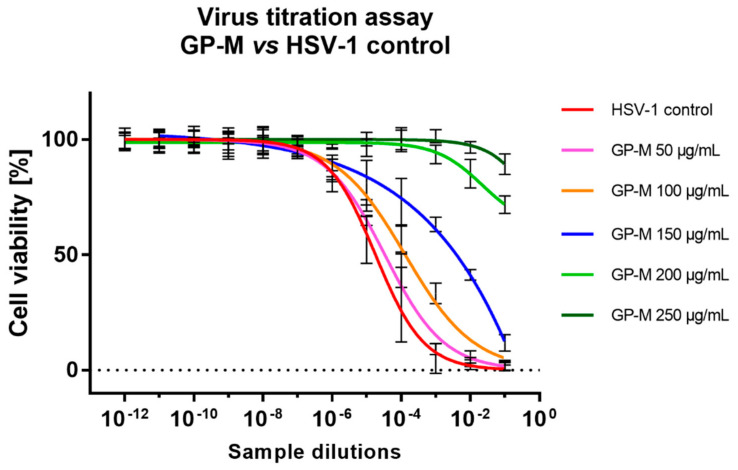
End-point dilution assay of HSV-1 titer in samples treated with *Geranium pyrenaicum* methanol extract.

The antiviral activity of *Geranium pyrenaicum* extracts was tested on HSV-1 infected VERO cells. As can be concluded form Figure 2, the hexane, ethyl acetate and aqueous extracts didn’t show any significant inhibition of cytopathic effect. However, the methanol extract in the concentrations of 250 and 200 µg/mL inhibited the formation of CPE. With decreasing concentration of GP-M (150, 100 and 50 µg/mL) an increase in the intensity of CPE could be observed. Subsequent measurement of viral infectious titer confirmed the lack of antiviral activity of GP-H, GP-EA, and GP-W with mean Δlog between −0.24 and 0.16 (logCCID_50_/mL) (Table 6). In case of GP-M in the concentration of 250 and 200 µg/mL the exact value of viral titer could not be evaluated (Figure 3) but the decrease in the viral titer was at least 4 log (logCCID_50_/mL). Whereas, for lower concentrations of GP-M—150, 100 and 50 µg/mL, a dose–response relationship could be observed with the reduction of viral titer by 2.1, 1.29, and 0.06 logCCID_50_/mL, respectively.

**Figure 4:**
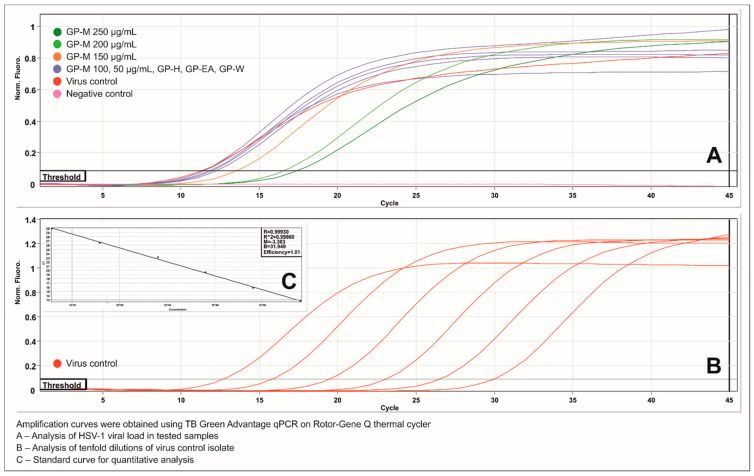
Real-Time PCR analysis of HSV-1 viral load in tested samples.

The GeneProof Herpes Simplex Virus (HSV-1/2) PCR Kit allowed for quantitative analysis of the HSV-1 viral load in the dilutions of virus control isolate prepared from virus infected VERO cell line, 72 h post infection. Subsequently, those dilutions were analyzed using TB Green Advantage qPCR Premixes to obtain a standard curve for further analysis (Figure 4). The Real-Time PCR analysis of DNA isolates from the samples collected throughout antiviral assays showed that in the virus control the viral load of HSV-1 was 6.23–6.57 log(copies/mL), whereas, in case of *Geranium pyrenaicum* hexane, ethyl acetate and aqueous extracts ranged between 6.07 and 6.29 log(copies/mL), confirming no influence on HSV-1 replication. However, for *Geranium pyrenaicum* methanol extract a dose–response relationship between the viral load and the tested concentration could be observed (Table 6). The GP-M in the concentrations of 250 and 200 µg/mL reduced the viral load by 1.72 and 1.55 log, respectively. Post-amplification melting curve analysis showed peaks at 85.3–85.5 °C for all samples. 

It was reported that *Geranium thunbergii* ethanol extract possessed antiviral activity towards influenza virus and the mechanism of action is probably due to the inhibition of neuraminidase (NA) activity. The NA is involved in increasing the mobility of influenza virus through the mucosa of respiratory tract and in the release of the virus progeny from the infected cells. The *Geranium thunbergii* ethanol extract inhibited NA activity of influenza type A (H1N1 (A/PR/8/34, and A/Korea/33/2005), H3N2 (A/Korea/32/2005)), and influenza type B (B/Korea/72/2006) and the activity was highest when 250 μg/mL concentration was used. Additionally, the inhibition of viral replication in H1N1 infected MDCK cells was observed with viral titers were reduced by 4.8 logTCID_50_/mL (TCID_50_—50% tissue culture infectious dose), and complete inhibition of influenza A HA (hemagglutinin) production and NS-1 mRNA expression was found [12]. Serkedjieva and Ivancheva [14] studied the anti-influenza virus activity of polyphenolic complex isolated from *Geranium sanguineum* L. and reported the inhibition of hemagglutinin (HA) expression on the surface of cells infected with influenza A/chicken/Rostock/34 (H7N1) strain, reduction of virus-induced cytopathic effect, infectious virus yield plaque formation, and decreased synthesis of viral proteins at non-toxic concentrations, suggesting significant antiviral activity [14]. Moreover, the antiviral activity of polyphenolic complex was also found for several other strains of influenza virus, ex. A/Victoria/36/88 (H1N1), A/Gabrovo/539/79 (H1N1), A/Krasnodar/101/59 (H2N2), A/Beijimg/352/89 (H3N2), and A/Sofia/92/72 (H3N2). Interestingly, higher concentrations of polyphenolic complex also exerted virucidal effects, resulting in reduction of HA titer 4-fold and plaque infectious titer by 1 log at the dose of 100 µg/mL, whereas at 200 µg/mL, the biological activity of the virus was abolished. These results suggest that early events during influenza virus replication cycle are the most probable target for polyphenolic complex isolated from *Geranium sanguineum* L. and their inhibition is responsible for the reported activity [14]. Aqueous extract obtained from aerial roots of *Geranium sanguineum* L. was found to exert antiviral and virucidal activity towards HSV-1 and HSV-2. Virucidal activity was observed towards HSV-1 at concentrations of above 100 µg/mL. The antiviral activity was observed at 32 µg/mL and decreased with lowering concentration of extract showing dose-response relationship. To evaluate which of the HSV-1 replication steps were inhibited the extract was added at different times relative to viral infection. Interestingly, pre-treatment of VERO cells as well as the addition at the time of adsorption had no effect on the virus titer. Noticeable reduction of HSV-1 titer was found when the extract was added at the time of penetration (32 µg/mL; ΔlogTCID_50_/mL = 1.0), whereas, significant reduction of viral titer was observed when the extract was introduced after virus infection (32 µg/mL; ΔlogTCID_50_/mL = 2.73). Moreover, the highest HSV-1 inhibition was expressed when the *Geranium sanguineum* L. aqueous extract was present in the growth medium during the whole replication cycle (32 µg/mL; ΔlogTCID_50_/mL = 3.5) [13]. In our study, the *Geranium pyrenaicum* methanolic extract decreased the HSV-1 titer by more than 4 log CCID_50_/mL, however, this activity was observed at much higher concentrations than those reported for *Geranium sanguineum* L. aqueous extract. Additionally, different solvents used for extraction (water vs. methanol) may suggest that diverse compounds may be responsible for antiviral activity of each *Geranium* species. It is also worth mentioning that *Geranium pyrenaicum* aqueous extract was deprived of any noticeable antiviral activity. 

To evaluate which active compound may be responsible for antiviral activity we have compared methanolic extract with those obtained using other solvents. It can be clearly seen that *Geranium pyrenaicum* methanolic extract shows presence of several compounds which are absent in other extracts, ex. 3-*O*-galloylquinic acid, dihydroxybenzoic acid glucoside, galloylshikimic acid, di-HHDP-glucose isomers 1 and 2 (Pedunculagin I structure), HDDP-galloyl-glucose isomer 1-(Corilagin structure), methyl brevifolincarboxylate derivative, methyl brevifolincarboxylate isomer 2, geraniin, dehydrochebulic acid trimethyl ester, kaempferol-*O*-galloylglucoside, ellagic acid-galloyl-glucoside derivative, HHDP-galloyl derivative, and kaempferol-3-*O*-rutinoside. It was reported that galloylquinic acids and galloylshikimic acid, especially tri- and tetragalloylquinic acids, and 3,5-Di-*O*-galloylshikimic acid, are potent HIV-RT (HIV reverse transcriptase) inhibitors [69,70]. Benzoic acid derivatives, especially 2,5-dihydroxybenzoic acid (gentisic acid), were recently found during molecular docking studies to be promising inhibitors of SARS-CoV-2 main protease [71]. Another molecular docking study reported pedunculagin among several other tannins, as a potent inhibitor of 3CL^pro^ (3-chymotrypsin-like cysteine protease) of SARS-CoV-2 [72]. Corilagin was found to possess antiviral activity towards human enterovirus 71 (EV71) and Coxsackievirus A16 [73], hepatitis C virus (HCV) [74], and additionally was shown to regulate the immune response during Herpes simplex encephalitis and relieve inflammatory injury by intruding with the TLR3 (toll-like receptor 3) signalling pathway [75]. The methyl brevifolin carboxylate was reported to inhibit influenza virus A/Puerto Rico/8/34 (H1N1) and A/Aichi/2/68 (H3N2) by targeting PB2 cap-binding domain [76]. Geraniin was found to exert broad spectrum antiviral activity, inhibiting HSV-2 [77], human enterovirus 71 [44], as well as Zika and Dengue viruses [78]. It can be concluded that the presence of the above mentioned bioactive antiviral molecules may be related to the anti HSV-1 activity of *Geranium pyrenaicum* methanolic extract.

A noticeable discrepancy can be observed when comparing the results of reduction of the infectious viral titer with the reduction of the viral load found for *Geranium pyrenaicum* methanol extract. In case of the infectious viral titer, the magnitude of inhibition was significantly higher, however, it must be underlined that those values are difficult to be directly compared. End-point dilution assay allows for measurement of the infectious titer, meaning that only the viral particles capable of infecting permissible cells will be titrated. Whereas, the Real-Time PCR will detect and quantify copies of a target DNA sequence specific for HSV-1 in the collected samples. This target DNA sequence may be found inside both infectious and non-infectious viral particles, as well as inside virus infected cells. Lower reduction of the viral load compared to infectious titer may be due to inhibition of the HSV-1 replication during production of viral proteins, as was suggested for benzoic acid derivatives and pedunculagin [71,72], when viral DNA synthesis has occurred but further replication was interrupted. When the plates were repeatedly frozen and thawed at the end of antiviral assays, the incomplete, non-infectious viral particles were released and the viral DNA measured using qPCR. Further studies will be needed to evaluate which of the HSV-1 replication steps was inhibited by the *Geranium pyrenaicum* methanol extract, and to identify compounds responsible for this activity. 

### 2.6. KEGG Pathway Enrichment Analysis of the Major Compounds

**Figure 5:**
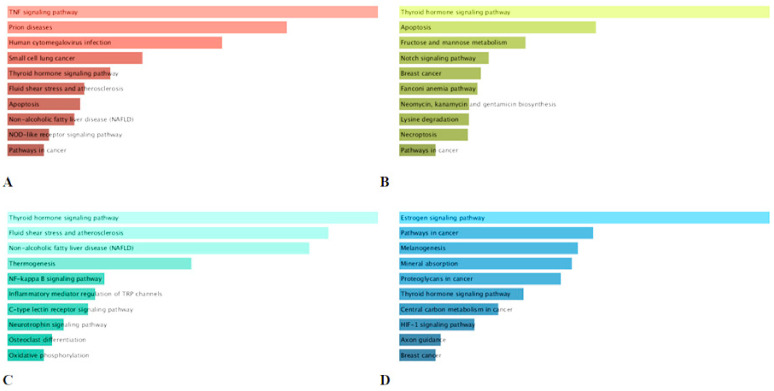
The top 10 KEGG pathway of modulated mRNA (C&D) by the major compounds of *Geranium pyrenaicum*. (**A**) Corilagin; (**B**) Pedunculagin; (**C**) Tellimagrandin I; (**D**) Ellagic acid.

The results of the KEGG pathway enrichment analysis of identified four major compounds of geranium was depicted in Figure 5. 19, 21, 67 and 321 mRNA were modulated by corilagin, pedunculagin, tellimagrandin I and ellagic acid respectively. With respect to the first enriched pathway, ‘TNF signaling pathway’, ‘thyroid hormone signaling pathway’ and ‘estrogen signaling pathway’ were found to be predict by the mRNAs targeted by corilagin, pedunculagin, tellimagrandin I and ellagic acid respectively (Figure 5A–D).

TNF (tumor necrosis factor) is an important mediator of apoptosis, immunity and inflammation, and it has been involved in the pathogenesis of a large spectrum of human illnesses such as cancer, diabetes, rheumatoid arthritis, osteoporosis, sepsis and inflammatory bowel diseases [79]. TNF activity regulators are being advance for the management of above-mentioned disease. Incidentally, corilagin have been shown to significantly downregulate TNF induced expression [80]. Similarly, several phenolic compounds from pants including eriodictyol, luteolin, quercetin, hesperetin, resveratrol and amoradicin have been observed to be potent inhibitors of the TNF activity [81]. Further, thyroid hormone (TH) have been reported to modulate multiple physiological processes such as cellular growth, embryonic development, metabolism, differentiation and proliferation [82]. Similarly, multiple physiological responses in several tissue types are regulated by estrogen hormone [83]. Moreover, TH regulates various extracellular and intracellular candidate proteins, whereas estrogen is an activator of protein synthesis, cytoplasmic kinase [82,84]. However, disruption of both hormones level and activity has been shown to play a key role in the pathogenesis and progression of several diseases. Notably, TH boosts cancer cell proliferation via disruption of molecular and signaling pathways [82]. Indeed, clinical hypothyroidism leads to retard cancer growth; in contrast, hyperthyroidism is associated with cancer prevalence in multiple tumor types i.e., breast, brain, thyroid, liver lung and colorectal cancer. Additionally, when thyroid hormone is out of control, it can cause diabetes mellitus, cardiovascular diseases [85]. Regarding estrogen, it plays crucial roles in the development and proliferation of breast cancers, diabetes, Alzheimer’s and cardiovascular diseases [84]. Thereby, potent modulator of TH and estrogen can be considered potential therapeutic agents against above mentioned disease. Polyphenols are able to bind to hormones receptor, and thereby induce biological effects in cells through inhibiting or mimicking the action of endogenous hormones [86]. Illustratively, many polyphenols including daidzein and genistein show stronger affinity for estrogen α and β receptors [87]. Kaempferol present in various plants has been associated with modulation of thyroid hormone signaling pathway [88]. In the light of our results, pedunculagin, tellimagrandin I and ellagic acid could act as both hormones agonists and antagonists and therefore they might be employed to sensitize cancer cells to chemotherapeutic drugs by modulating pathways that cause treatment resistance.

## 3. Materials and Methods

### 3.1. Plant Material and Preparation of Extracts

*Geranium pyrenaicum* was collected (Hanönü village, Kastamonu, Turkey) in 2020, and authentication was performed by one of the co-authors (Dr. Ismail Senkardes). One voucher specimen was kept in the herbarium of Pharmacy Faculty, Marmara University (Voucher no: MARE-19858). The plant species (aerial parts) were dried in well-ventilated and dark condition for ten days. The dried aerial parts were powdered by using one laboratory mill. The plant materials were extracted with different solvents including hexane, ethyl acetate, methanol and water. For organic solvents, the plant materials (10 g) were macerated with 200 mL of solvent at room temperature for 24 h. The filtered and they concentrated by using one rotary-evaporator under vacuum. Water extract was prepared with traditional infusion technique and the plant materials (10 g) were waited in the boiled water (200 mL) for 15 min. Then, the extract filtered and lyophilized. All extracts were stored in +4 °C until analysis. 

### 3.2. Profile of Bioactive Compounds

Traditional spectrophotometric methods were used to evaluate total amounts of phenolics and flavonoids in the tested extracts. The details for the experiments were reported in our previous papers and can be found in the Supplementary Materials. Gallic acid (GAE) and rutin (RE) were used as standards in the assays [89,90]. 

### 3.3. LC-ESI-QTOF-MS/MS Analysis

The phytochemical analysis of studied extracts was done by means of liquid chromatography coupled to mass spectrometry (LC-ESI-QTOF-MS/MS) on Agilent 1200 Infinity HPLC and Agilent 6530B QTOF (Agilent Technologies, Santa Clara, CA, USA) supported by high purity nitrogen generator (Parker Hannifin Corporation, Haverhill, MA, USA). The chromatographic conditions and spectroscopic parameters followed our previous paper [91]. Compounds identification was based on their fragmentation patterns and supported by comparison of obtained mass spectra with mass spectra available in Metlin database (https://metlin.scripps.edu, accessed on 15 April 2021), MassBank of North America (MoNA) database (https://mona.fiehnlab.ucdavis.edu/, accessed on 15 April 2021), Human Metabolome Database (HMDB) (https://hmdb.ca/, accessed on 15 April 2021), PubChem—an open chemistry database (https://pubchem.ncbi.nlm.nih.gov/, accessed on 15 April 2021) and literature data.

### 3.4. Determination of Antioxidant and Enzyme Inhibitory Effects

Antioxidant properties of *Geranium* extracts were tested by different chemical assays including radical scavenging, reducing power and metal chelating. Trolox (TE) and EDTA (EDTAE) were selected as standard antioxidants for evaluating antioxidant properties. Enzyme inhibitory properties were assayed against different enzymes such as tyrosinase, α-amylase, α-glucosidase and cholinesterases. Kojic acid (for tyrosinase), acarbose (for amylase and glucosidase) and galantamine (for acetylcholinesterase and butrylcholinesterase) [92]. The details for the experiments can be found in the Supplementary Materials.

### 3.5. Cell Line Maintenance and Sample Preparation for In Vitro Assays

The Vero cells were cultured using Dulbecco Modified Eagle Medium (DMEM, Corning, Tewksbury, MA, USA), and Modified Eagle Medium (MEM, Corning) was used for cancer cell lines. All media was supplemented with penicillin and streptomycin (Penicillin-Streptomycin Solution, Corning) and fetal bovine serum (FBS, Capricorn)—10% for cell passaging and 2% for cell maintenance and experiments. The media for MTT was FBS free. Phosphate buffered saline (PBS) and trypsin were obtained from Corning, whereas, 3-(4,5-dimethylthiazol-2-yl)-2,5-diphenyltetrazolium bromide (MTT) and dimethyl sulfoxide (DMSO) from Sigma (Sigma-Aldrich, St. Louis, MO, USA). Cells were incubated in 5% CO_2_ atmosphere at 37 °C (CO_2_ incubator, Panasonic Healthcare Co., Tokyo, Japan). *Geranium pyrenaicum* extracts were dissolved (50 mg/mL) in DMSO and membrane filtered (0.2 µm) to obtain stock solutions used in experiments. Stock solutions were stored frozen until used. 

### 3.6. Cytotoxicity Assessment

For cell cytotoxicity assay the cells were passaged to 96-well plates (Falcon, clear flat bottom TC-treated, Corning) and incubated for 24 h. Afterwards, a semi confluent monolayer of appropriate cell line was treated with serial dilutions of extract stock solutions (1000–0.49 µg/mL) for 72 h. Simultaneously, the influence of DMSO, used as a solvent for stock solutions, on the cells was evaluated. Cells supplemented with 2% FBS culture media were used as a negative control. After incubation the media was removed, PBS was used to wash the cells and 10% of MTT solution (5 mg/mL) in FBS free media was added and the incubated for the next 4 h. Subsequently, the SDS/DMF/PBS (14% SDS, 36% DMF, 50% PBS) solvent was added (100 µL per well) to solubilize the formasane crystals and after overnight incubation the Synergy H1 Multi-Mode Microplate Reader (BioTek Instruments, Inc., Winooski, VT, USA) equipped with Gen5 software (ver. 3.09.07; BioTek Instruments, Inc.) was used for absorbance measurement (540 and 620 nm). The in vitro test were done in triplicate and repeated three times. Data analysis was performed using GraphPad Prism (v8.0.1) to calculate CC_50_ (concentration decreasing cell viability by 50% in comparison to control cells) from dose–response curves (non-linear regression). Furthermore, the selectivity indexes were calculated (VERO CC_50_/cancer cell line CC_50_) to evaluate the selectivity towards cancer cells. In case of VERO cells also CC_10_ (concentration decreasing cell viability by 10% in comparison to control cells) values were evaluated for the use in further antiviral studies. Statistical evaluation was performed using GraphPad Prism (two-way ANOVA, Dunnett’s multiple comparisons test).

### 3.7. Evaluation of Antiviral Activity—Influence on CPE Formation

The evaluation of antiviral activity was carried out against HSV-1 propagated in VERO cell line. The infectious titer of HSV-1 was 5.5 ± 0.25 logCCID_50_/mL (CCID_50_—50% cell culture infectious dose). Briefly, the VERO cells were passaged into 48-well plates (Falcon, clear flat bottom TC-treated, Corning, Tewksbury, MA, USA) and incubated for 24 h to produce monolayer. Afterwards, the cells were treated with 500 µL/well of HSV-1 suspension (100 * CCID_50_/mL) in cell media and incubated for 1 h. Uninfected cells were left as controls. After incubation the media were removed from infected wells and after washing with PBS the extracts diluted in cell media in non-toxic concentrations (concentrations selected based on the CC_10_ values) were added. The cell control and virus control wells were supplemented with media containing 2% FBS. The plates were incubated until cytopathic effect (CPE) was observed (inverted microscope CKX41, Olympus Corporation, Tokyo, Japan) in virus control. Afterwards, the inhibition of CPE by tested samples in comparison with the virus control was recorded. Finally, the plates were frozen and thawed thrice, the samples were collected, and kept in −72 °C until used in end-point virus titration assay and viral DNA isolation. Antiviral properties of extracts were tested in three independent experiments.

### 3.8. End-Point Dilution Assay for HSV-1 Titration

To perform the end-point virus titration assay the VERO cells were passaged into 96-well plates and incubated for 24 h to produce monolayer. Afterwards, the ten-fold dilutions of samples collected during evaluation of antiviral activity in cell media were prepared and incubated with cells for 72 h. The plates were observed using inverted microscope and the observations recorded daily. Finally, after the incubation, media were removed from plates and the HSV-1 infectious titer in all samples was measured using the MTT method as described above. The measure of antiviral activity was the difference (Δlog) of HSV-1 infectious titer (logCCID_50_/mL) in the samples collected from tested extracts (TE) in comparison with the virus control (VC) from the same experiment (Δlog = logCCID_50_/mL VC–logCCID_50_/mL TE). The end-point virus titration was performed for every antiviral assay and the results are the mean of viral titer from all experiments. A significant antiviral activity can be reported for extracts decreasing the infectious titer by at least 3 log compared to virus control.

### 3.9. Viral DNA Isolation and Real-Time PCR Analysis

The DNA isolation was carried out using QIAamp DNA Mini Kit (Cat#51304, QIAGEN GmbH, Hilden, Germany) according to manufacturer’s protocol. The Real-Time PCR (qPCR) amplification was performed on Rotor-Gene Q (QIAGEN) thermal cycler. A series of ten-fold dilutions of the virus control isolate was prepared and the quantitative analysis of the virus copies was performed using IVD certified GeneProof Herpes Simplex Virus (HSV-1/2) PCR Kit (Cat#HSV/ISEX/025, GeneProof a.s., Brno, Czech Republic). The amplification conditions were as follows—hot start activation and initial denaturation (37 °C, 2 min; 95 °C, 10 min); cycling (45 repeats: denaturation (95 °C, 5 s), annealing (60 °C, 40 s), fluorescence acquisition (Green, Red, Yellow), extension (72 °C, 20 s).

The qPCR analysis of viral load in the tested samples was carried out using TB Green Advantage qPCR Premixes (Takara Bio, Mountain View, CA, USA) and primers (UL54F—5′ CGCCAAGAAAATTTCATCGAG 3′, UL54R—5′ ACATCTTGCACCACGCCAG 3′) for UL54 coding region (encoding ICP27—regulatory protein required for HSV-1 infection). The amplification conditions were as follows—initial denaturation (95 °C, 20 s); cycling (45 repeats: denaturation (95 °C, 5 s), annealing/extension (60 °C, 30 s), fluorescence acquisition (Green)); melting curve analysis (60–95 °C). The HSV-1 titer in samples was assessed with reference to a calibration curve produced from ten-fold dilutions of the virus control isolate previously analyzed using GeneProof diagnostic kit.

### 3.10. Statistical Analysis

All the antioxidant and enzyme inhibitory activity experiments were done in triplicate and the results were given as means ± standard deviation. Comparison between the samples was achieved using One way ANOVA followed by Tukey’s multiple range test (*p* < 0.05). Xlstat (Addinsoft, New York, NY, USA) was used for the data analysis. 

### 3.11. Bioinformatics Analysis

Datasets for mRNA from DIGEP-Pred web-sever [93] was employed to explore the gene targets of the major molecules of including corilagin, pedunculagin, tellimagrandin I and ellagic acid. The genes with Pa (probability “to be active”) above 0.5 were used. Both upregulated and downregulated mRNA data were subjected to Enrichr websever [94] for KEGG pathway analysis. 

## 4. Conclusions

Investigation of the bio compounds of *G. pyrenaicum* presented in this paper contributes to the knowledge on the phytochemistry of this plant and advances the knowledge on its antioxidant and enzyme inhibitory effects. Aqueous extract of the plant exhibited the strongest antioxidant activity with most assays correlating with its high TPC. Among the four tested extracts, the ethyl acetate and methanolic extracts showed high enzyme inhibitory effects. Data gathered from this study claimed that compounds such as 3-*O*-galloylquinic acid, dihydroxybenzoic acid glucoside, galloylshikimic acid, di-HHDP-glucose isomers 1 and 2 (Pedunculagin I structure), HDDP-galloyl-glucose isomer 1-(Corilagin structure), methyl brevifolin carboxylate derivative, methyl brevifolin carboxylate isomer 2, geraniin, dehydrochebulic acid trimethyl ester, kaempferol-*O*-galloylglucoside, ellagic acid-galloyl-glucoside derivative, HHDP-galloyl derivative, and kaempferol-3-*O*-rutinoside identified in the methanolic extract of the plant were responsible in exhibiting anti HSV-1 activity. However, further research including clinical in vivo studies is recommended to further evaluate these aforementioned properties, in order to include these traditional plants as a potential pharmacological ingredient.

## Figures and Tables

**Table 1:** Total bioactive compounds and total antioxidant capacity (by phosphomolybdenum assays) of the tested extracts.

Solvents	TPC (mg GAE/g)	TFC (mg RE/g)	PBD (mmol TE/g)
Hexane	25.03 ± 0.27 ^d^	28.26 ± 1.61 ^c^	1.59 ± 0.07 ^c^
EA	31.25 ± 3.15 ^c^	43.95 ± 0.50 ^a^	2.13 ± 0.11 ^b^
MeOH	133.22 ± 0.34 ^b^	38.66 ± 0.73 ^b^	3.08 ± 0.15 ^a^
Water	170.50 ± 0.46 ^a^	25.11 ± 0.31 ^d^	3.15 ± 0.09 ^a^

Values are reported as mean ± SD. EA: Ethyl acetate; MeOH: Methanol; TPC: Total phenolic content; TFC: Total flavonoid content; PBD: Phosphomolybdenum; GAE: Gallic acid equivalent; RE: Rutin equivalent; TE: Trolox equivalent. Different letters in same column indicate significant differences in the tested extracts (*p* < 0.05).

**Table 2:** Chemical composition of the tested extracts.

Comp. No	Tentative Identification	Retention Time	Molecular Formula	Molecular Weight	[M-H]^−^	Fragments (m/z)	Extracts	References
1	Caffeic acid glucoside	1.520	C_12_H_22_O_11_	342.1109	341.1109	179.0572; 161.0482; 135.0455	1, 2, 3, 4	[16]
2	Quinic acid derivative	1.654	-	534.1755	533.1755	191.0583; 173.0415; 111.0477	3, 4	[16]
3	Quinic acid	1.771	C_7_H_12_O_6_	192.0584	191.0584	173.0184; 111.0078; 93.0373; 85.0305	1, 2, 3, 4	[17]
4	Malic acid	1.917	C_4_H_6_O_5_	134.0154	133.0154	115.0046; 89.268; 71.0147	1, 2, 3, 4	[18]
5	Citric acid	2.246	C_6_H_8_O_7_	192.0209	191.0209	111.0131; 87.0128; 57.0408	1, 3, 4	[19,20]
6	3-*O*-Galloylquinic acid	2.658	C_14_H_16_O_10_	344.0695	343.0695	191.0516; 169.0091; 125.0179; 111.0505 107.0141	3	[21]
7	Glucogallic acid/Glucosyl gallate	2.900	C_13_H_16_O_10_	332.0694	331.0694	271.0432; 211.0585; 169.0143; 151.0054; 125.0211;	1,2,3	[22]
8	Gallic acid	4.049	C_7_H_6_O_5_	170.0158	169.0158	125.0247; 106.9624; 83.0489; 79.0247; 51.0242	2, 3, 4	[17]
9	4-*O*-Galloylquinic acid	5.143	C_14_H_16_O_10_	344.0695	343.0695	191.0550; 173.0496; 169.0131; 125.0298; 85.0290	1, 2, 3, 4	[21]
10	Dihydroxybenzoic acid glucoside	6.337	C_13_H_16_O_9_	316.0748	315.0748	153.0217; 108.0278	3	[18,20,22]
11	Glycerol gallate	6.454	C_10_H_12_O_7_	244.0508	243.0508	169.0111; 125.0275; 124.0175	3	[17]
12	Dihydroxybenzoic acid	7.476	C_7_H_6_O_4_	154.0208	153.0208	109.0302; 108.0203; 91.0152; 53.0379	2, 3, 4	[18]
13	Gallic acid *O*-(6-galloylglucoside)/Di-galloyl-hexoside	8.103	C_20_H_20_O_14_	484.0812	483.0812	331.0590; 313.0453; 271.0677; 169.0110; 150.9913; 125.0221	3, 4	[17,23,24]
14	2-Isopropylmalic acid	8.243	C_7_H_12_O_5_	176.0614	175.0614	157.0537; 131.0713; 115.0409; 113.0609; 85.0661	3, 4	[18,25]
15	Galloylshikimic acid	9.056	C_14_H_14_O_9_	326.0585	325.0585	173.0437; 169.0163; 137.0592; 125.0260	3	[25,26,27]
16	di-HHDP-glucose isomer 1 (Pedunculagin I structure)	9.121	C_34_H_24_O_22_	784.0720	783.0720	481.0677; 301.0031; 275.0225; 249.0320; 169.0301	3	[28,29]
17	Dihydrocaffeic acid	10.037	C_9_H_10_O_4_	182.0157	181.0157	137.0283; 109.0356;	3, 4	[18]
18	Unknown	10.592	-	442.1640	441.1640	377. 1378; 317.1239; 275.1158; 233. 1032; 173.0836; 119.0361	1,2,3	
19	Unknown	10.636	-	452.2188	451.2188	405.1966	1, 2, 3, 4	
20	Methyl gallate	10.832	C_8_H_8_O_5_	184.0315	183.0315	168.0088; 124.0184; 78.0135	1, 2, 3, 4	[22]
21	di-HHDP-glucose isomer 2 (Pedunculagin I structure)	12.438	C_34_H_24_O_22_	784.0692	783.0692	481.0698; 301.0021; 275.0229; 249.0429; 169.0448	3	[27,28,29]
22	Caffeoylmalic Acid	13.375	C_13_H_12_O_8_	296.0480	295.0480	251.0566; 219.0290	3, 4	[20]
23	Benzyl alcohol *D*-xylopyranosyl *D*-glucopyranoside	14.119	C_18_H_26_O_10_	402.1448	447.1522 [M+HCOOH]^−^	401.1448; 269.1076; 161.0426; 149.0446	2, 3, 4	[20]
24	HDDP-galloyl-glucose isomer 1- (Corilagin structure)	14.299	C_27_H_22_O_18_	634.0689	633.0689	463.0484; 300.9942; 275.0090; 169.0082	3	[27,30,31]
25	Di-galloylo-quinic acid	15.179	C_21_H_20_O_14_	496.0805	495.0805	343.0615; 191.0564; 169.0102	3, 4	[32]
26	Methyl brevifolincarboxylate derivative	15.897	-	382.0480	381.0480	337.0593; 305.0301; 273.0018; 261.0420	3	[20]
27	Brevifolin	17.761	C_10_H_12_O_4_	248.0268	247.0268	219.0324; 191.0495; 173.0356; 145.0434; 117.0400	3, 4	[20,33,34]
28	Brevifolincarboxylic acid	18.026	C_13_H_8_O_8_	292.0169	291.0169	247.0269; 191.0358; 173.0274; 163.0419; 145.0339	3, 4	[20,32,35]
29	HDDP-galloyl-glucose isomer 2 (Corilagin structure)	19.130	C_27_H_22_O_18_	634.0768	633.0768	463.0507; 300.9996; 275.0242; 245.0085; 169.0145; 125.0232	3, 4	[27,30,31]
30	di-galloyl-HHDP-glucoside isomer 1 (Tellimagrandin I structure)	19.268	C_34_H_26_O_22_	786.0725	785.0725	633.0786; 615.0878; 483.0649; 300.9964; 275.0119; 249.0340; 169.0128I	3, 4	[30]
31	Geraniin	20.417	C_41_H_27_O_27_	952.0548	951.0548	933.0557; 915.0390; 802.8552; 463.0388; 300.9928; 169.0060	3	[31,36]
32	Methyl brevifolincarboxylate isomer 1	20.886	C_14_H_10_O_8_	306.0325	305.0325	273.0017; 245.0049; 217.0084; 173.0185; 161.0211; 145.0259; 133.0261; 117.0297; 105.0318	3, 4	[33,35]
33	Dehydrochebulic acid trimethyl ester	22.134	C_17_H_16_O_11_	396.0647	395.0647	363.0370; 351.0818; 319.0430; 287.0224	3	[20,37]
34	Shikimic acid derivative	22.992	-	296.0462	295.0462	173.0056; 154.9935; 129.0224; 111.0080	3, 4	[20]
35	Methyl brevifolincarboxylate isomer 2	23.398	C_14_H_10_O_8_	306.0312	305.0312	273.0026; 245.0060; 217.0143; 201.0204; 189.0203; 173.0235; 161.0230; 145.0310; 133.0298;	3	[33,35]
36	Ferulic acid	23.563	C_10_H_10_O_4_	194.0509	193.0509	178.0311; 149.0628; 135.0424; 134.0364; 106.0464	3, 4	[18]
37	Quercetin-*O*-hexoside derivative	23.773	-	774.1598	773.1598	463.0868; 301.0189; 300.0253; 193.0506	3, 4	[18]
38	Quercetin-*O*-hexoside	23.900	C_21_H_20_O_12_	464.0909	463.0909	301.0241; 300.0241; 271.0248; 255.0258 178.9943; 151.0021	1, 2, 3, 4	[18]
39	Ellagic acid	24.240	C_14_H_6_O_8_	302.0022	301.0022	300.9990; 283.9952; 245.0101; 229.0198; 200.0098; 173.0274; 157.2608; 161.0196; 145.0282	3, 4	[18,38]
40	Nonanedioic acid	24.305	C_9_H_16_O_4_	188.0981	187.0981	169.0836; 143.0994; 125.0960; 97.0642	2	[18]
41	Quercetin-*O*-glucuronide	24.569	C_21_H_18_O_13_	478.0684	477.0684	301.0353; 178.9961; 151.0043	3, 4	[38,39]
42	Quercetin-*O*-(galloyl)-glucoside	24.856	C_28_H_24_O_16_	616.1008	615.1008	301.0359; 179.0011; 151.0061	2, 3, 4	[40]
43	Kaempferol-*O*-galloylglucoside	25.318	C_28_H_24_O_15_	600.1022	599.1022	447.0780; 313.0565; 285.0410; 284.0369; 255.0288; 169.0141; 151.0032; 125.0251	3	[18,41]
44	Kaempferol-*O*-glucoside	25.640	C_21_H_20_O_11_	448.0974	447.0974	285.0379; 284.0314; 255.0266; 151.0010	3, 4	[18,41]
45	HHDP-galloyl glucovanillin	26.653	C_35_H_28_O_20_	768.1081	767.1081	615.1026; 465.0779; 313.0672; 169.0220; 125.0308	3, 4	[20,27]
46	Ellagic acid-galloyl-glucoside derivative	28.253	-	998.1205	997.1205	827.0874; 615.0581; 463.0464; 300.9983; 169.0161	3	[27]
47	HHDP-galloyl derivative	28.467	-	752.1191	751.1191	599.1070; 465.0743; 313.0625; 169.0130; 125.0217	3	[27]
48	Quercetin	30.914	C_15_H_10_O_7_	302.0022	301.0022	257.0624; 178.9998; 151.0052	1, 2, 3, 4	[18]
49	Kaempferol-3-*O*-rutinoside	30.969	C_27_H_30_O_15_	594.1334	593.1334	285.0414; 284.0318; 255.0296; 151.0048	3	[33]
50	Luteolin	31.018	C_15_H_10_O_6_	286.0428	285.0428	199.0523; 175.0417; 149.0235; 133.0306	1, 2, 3, 4	[18]
51	Fatty acid	31.965	C_18_H_32_O_5_	328.2197	327.2197	-	1, 2, 3, 4	
52	Fatty acid	33.680	C_18_H_34_O_5_	330.2349	329.2349	-	1, 2, 3, 4	
53	Fatty acid derivative	37.072	-	536.2399	581.2461 [M+HCOOH]^−^	-	1, 2, 3, 4	
54	Unknown	37.698	-	308.1939	307.1939	289.1870; 235.1350; 185.1188; 121.0060	1, 2, 3, 4	
55	Fatty acid derivative	38.986	-	550.2535	595.2635 [M+HCOOH]^−^	-	1, 2, 3, 4	
56	Fatty acid derivative	41.062	-	564.2696	609.2767 [M+HCOOH]^−^	-	1, 2, 3, 4	

1—Hexane; 2—Ethyl acetate; 3—Methanol; 4—Water.

**Table 3:** Antioxidant properties of the tested extracts.

Solvents	DPPH (mg TE/g)	ABTS (mg TE/g)	CUPRAC (mg TE/g)	FRAP (mg TE/g)	MCA (mg EDTAE/g)
Hexane	na	6.02 ± 0.67 ^d^	64.18 ± 2.37 ^d^	30.68 ± 0.18 ^d^	12.13 ± 0.25 ^d^
EA	6.23 ± 0.53 ^c^	22.43 ± 1.74 ^c^	89.59 ± 1.07 ^c^	40.43 ± 0.29 ^c^	25.84 ± 2.26 ^c^
MeOH	199.26 ± 0.13 ^a^	448.84 ± 3.67 ^b^	514.79 ± 15.17 ^b^	294.54 ± 4.00 ^b^	36.53 ± 1.10 ^b^
Water	191.20 ± 0.18 ^b^	469.82 ± 0.34 ^a^	613.27 ± 4.64 ^a^	364.10 ± 1.71 ^a^	52.39 ± 0.15 ^a^

Values are reported as mean ± SD. EA: Ethyl acetate; MeOH: Methanol; TE: Trolox equivalent; EDTAE: EDTA equivalents; na: not active. Different letters in same column indicate significant differences in the tested extracts (*p* < 0.05).

**Table 4:** Enzyme inhibitory effects of the tested extracts.

Solvents	AChE (mg GALAE/g)	BChE (mg GALAE/g)	Tyrosinase (mg KAE/g)	Amylase (mmol ACAE/g)	Glucosidase (mmol ACAE/g)
Hexane	3.41 ± 0.09 ^b^	10.05 ± 0.13 ^ab^	113.33 ± 0.37 ^b^	0.92 ± 0.01 ^b^	2.23 ± 0.04 ^b^
EA	4.49 ± 0.09 ^a^	12.26 ± 0.98 ^a^	109.11 ± 0.65 ^c^	1.04 ± 0.03 ^a^	2.18 ± 0.01 ^b^
MeOH	4.39 ± 0.30 ^a^	8.49 ± 1.60 ^b^	121.42 ± 0.33 ^a^	0.87 ± 0.03 ^b^	2.39 ± 0.03 ^a^
Water	0.65 ± 0.09 ^c^	na	28.53 ± 1.11 ^d^	0.21 ± 0.01 ^c^	2.04 ± 0.03 ^c^

Values are reported as mean ± SD. EA: Ethyl acetate; MeOH: Methanol; GALAE: Galatamine equivalent; KAE: Kojic acid equivalent; ACAE: Acarbose equivalent; na: not active. Different letters in same column indicate significant differences in the tested extracts (*p* < 0.05).

**Table 5:** Cytotoxicity of *Geranium pyrenaicum* extracts.

*Geranium pyrenaicum*		VERO		FaDu		HeLa		RKO	
Solvent	Sample Code	CC_50_	CC_10_	CC_50_	SI	CC_50_	SI	CC_50_	SI
Hexane	GP-H	76.07 ± 3.63	40.1 ± 9.06	75.46 ± 6.40	1.0	73.28 ± 3.37	1.0	53.72 ± 2.07	1.4
Ethyl acetate	GP-EA	46.53 ± 1.61	15.75 ± 3.20	39.49 ± 5.18	1.2	32.34 ± 1.27	1.4	35.27 ± 1.14	1.3
Methanol	GP-M	481.50 ± 47.09	222.61 ± 35.1	66.92 ± 8.0 *	7.2	132.44 ± 11.22 *	3.6	124.77 ± 14.79 *	3.9
Water	GP-W	435.93 ± 32.31	36.23 ± 5.90	40.22 ± 2.89 *	10.8	63.21 ± 8.67 *	6.9	96.27 ± 13.72 *	4.5

CC_50_—50% cytotoxic concentration (µg/mL (mean ± SD)); CC_10_—10% cytotoxic concentration (µg/mL (mean ± SD)); SI—selectivity index (VERO CC_50_/cancer cell line CC_50_); *—statistically highly significant (*p* < 0.001). Statistical significance based on two-way ANOVA (Dunnett’s multiple comparisons test, 95% confidence interval) calculated for results obtained on cancer cells with reference to VERO cells.

**Table 6:** Reduction of HSV-1 infectious titer and viral load by the *Geranium pyrenaicum* extracts.

*Geranium pyrenaicum* Extracts				
Solvent	Sample Code	Concentration (µg/mL)	Reduction of HSV-1 Infectious Titer (Δlog) *	Reduction of HSV-1 Viral Load (Δlog’) **
Hexane	GP-H	40	0.09 ± 0.28	0.17 ± 0.19
Ethyl acetate	GP-EA	15	−0.24 ± 0.39	0.17 ± 0.18
Methanol	GP-M	250	>4	1.72 ± 0.19
		200	>4	1.56 ± 0.04
		150	2.1 ± 0.25	0.64 ± 0.06
		100	1.29 ± 0.17	0.23 ± 0.04
		50	0.06 ± 0.27	0.32 ± 0.03
Water	GP-W	40	0.16 ± 0.18	0.24 ± 0.11

* Δlog (mean ± SD)—mean was calculated from at least three titration assays from different antiviral assays. Δlog = logCCID_50_VC–logCCID_50_TE; VC—virus control; TE—tested extract, Δlog of at least 3 is regarded significant. ** Δlog’ (mean ± SD)—mean was calculated from two qPCR runs from different antiviral assays. Δlog’ = log(copies/mL)VC–log(copies/mL)TE; VC—virus control; TE—tested extract.

## Supplementary Materials

The following are available online at https://www.mdpi.com/article/10.3390/ijms22147621/s1. Reference [95] is cited in the supplementary materials.
